# Development and Application of a Total Diet Quality Index for Toddlers

**DOI:** 10.3390/nu13061943

**Published:** 2021-06-05

**Authors:** Melissa C. Kay, Emily W. Duffy, Lisa J. Harnack, Andrea S. Anater, Joel C. Hampton, Alison L. Eldridge, Mary Story

**Affiliations:** 1 Duke Global Health Institute, Duke University, Durham, NC 27708, USA; mary.story@duke.edu; 2 Gillings School of Global Public Health, University of North Carolina, Chapel Hill, NC 27599, USA; ebwelker@email.unc.edu; 3 School of Public Health, University of Minnesota, Minneapolis, MN 55454, USA; harna001@umn.edu; 4 Research Triangle Institute International, Research Triangle Park, NC 27709, USA; aanater@rti.org (A.S.A.); jhampton@rti.org (J.C.H.); 5 Nestlé Research, Nestlé Institute of Health Sciences, Route du Jorat 57, 1000 Lausanne, Switzerland; Alison.Eldridge@rdls.nestle.com

**Keywords:** diet quality, toddlers, dietary guidelines, dietary recommendations, eating patterns, Healthy Eating Index, child nutrition

## Abstract

For the first time, the 2020–2025 Dietary Guidelines for Americans include recommendations for infants and toddlers under 2 years old. We aimed to create a diet quality index based on a scoring system for ages 12 to 23.9 months, the Toddler Diet Quality Index (DQI), and evaluate its construct validity using 24 h dietary recall data collected from a national sample of children from the Feeding Infants and Toddlers Study (FITS) 2016. The mean (standard error) Toddler DQI was 49 (0.6) out of 100 possible points, indicating room for improvement. Toddlers under-consumed seafood, greens and beans, and plant proteins and over-consumed refined grains and added sugars. Toddler DQI scores were higher among children who were ever breastfed, lived in households with higher incomes, and who were Hispanic. The Toddler DQI performed as expected and offers a measurement tool to assess the dietary quality of young children in accordance with federal nutrition guidelines. This is important for providing guidance that can be used to inform public health nutrition policies, programs, and practices to improve diets of young children.

## 1. Introduction

Proper nutrition during infancy and early childhood is foundational for good health, with both short- and long-term implications. In the short term, proper nutrition influences optimal physical and cognitive growth and development. Over the long term, nutrition is important for reducing risk of diet-related chronic diseases and obesity [1,2]. Food preferences and dietary patterns are established during infancy and early childhood, setting the stage for healthy (or unhealthy) eating habits later in life [3,4]. For example, eating more fruits and vegetables during infancy is associated with higher fruit and vegetable preference and consumption at age 6 years [5]. However, unhealthy dietary behaviors during early childhood, such as sugar-sweetened beverage (SSB) consumption, can lead to increased consumption during later childhood and increased risk for obesity that continues into adulthood [6,7]. Therefore, unhealthy or sub-optimal dietary patterns should be identified and addressed in early childhood to mitigate the risk of both current and future health concerns and to promote healthy eating habits early in life.

Since 1980, The Dietary Guidelines for Americans (DGAs) have been jointly updated and issued every five years by the U.S. Departments of Agriculture and Health and Human Services [8]. However, until recently, the DGAs did not include recommendations for children under 2 years of age. In December 2020, the 2020–2025 DGAs were released and included guidance for healthy dietary patterns by life stage, including for children under the age of 2 years [9]. Diet quality assessments can evaluate how closely aligned eating patterns are with dietary recommendations. A commonly used tool to evaluate the degree to which a person’s diet aligns with the DGAs is called the Healthy Eating Index (HEI) [10]. The most recent HEI, the HEI-2015, is a valid and reliable metric used to assess how well a set of foods and beverages align with the 2015–2020 DGAs [11,12]. The HEI-2015 has been used for children ages 2–18 years, but a metric to assess diet quality similar to the HEI does not exist for infants and toddlers [13].

As evidenced in a recent systematic review, few studies have assessed the total diet quality among U.S. infants and toddlers, in part because of limited indices appropriate for this young population group [14]. Some studies use indices originally designed to assess diet quality in older populations and may not appropriately evaluate the diets of young children [15]. Other studies use a diet diversity score as a measure of diet quality [16]. However, these indices are generally used to estimate risk for nutrient deficiencies. They also rely on a limited selection of components and use arbitrary cutoffs [17]. Evaluating the diet as a whole is important because often more of one food or beverage means less of another and the overall combination informs the comprehensive nutritional status of the child. Other studies create their own definitions for “healthy” and “unhealthy” dietary patterns to assess diet quality. Hamner and Moore assessed the diet quality of children aged 6 months to 4 years using a modified Diet Quality Index Score [18]. However, without specific dietary guidelines to base scoring on, the index had a limited set of components with wide scoring ranges.

One of the recommended research needs from the 2020–2025 Scientific Report of the Dietary Guidelines Advisory Committee, an independent committee of nationally recognized experts convened to guide the development of the 2020–2025 DGAs, is to develop a dietary pattern scoring system, such as the HEI, for children from birth to less than 24 months [19]. Thus, the purpose of this study was threefold: (1) to create a Toddler Diet Quality Index (DQI) for children ages 12 to 23.9 months using evidence-based guidelines and dietary recommendations from the newly released 2020–2025 DGAs; (2) to evaluate the construct validity of the Toddler DQI using 24 h dietary recall data collected from a national sample of U.S. children from the Feeding Infants and Toddlers Study (FITS) 2016 and compare diet quality scores across demographic characteristics and breastfeeding behaviors; and (3) to describe the dietary quality of toddlers, thereby providing guidance that can be used to inform public health nutrition policies, programs, and practices.

## 2. Methods

### 2.1. Diet Quality Index Development

The HEI-2015 was used as a guiding framework for development of the Toddler DQI described in this paper [12]. The HEI is a tool developed by the U.S. Department of Agriculture and the National Cancer Institute to determine conformance with the DGAs. The HEI-2015 consists of 13 components, 9 of which assess adequacy of the diet, including (1) total fruit; (2) whole fruit; (3) total vegetables; (4) greens and beans; (5) whole grains; (6) dairy; (7) total protein foods; (8) seafood and plant proteins; and (9) fatty acids, which is a ratio of unsaturated versus saturated fatty acids. The remaining assess dietary components to limit: (10) refined grains; (11) sodium; (12) added sugars; and (13) saturated fat. Higher scores reflect better diet quality as moderation components are reverse scored. Each component is scored on a density basis, either as a percentage of calories or per 1000 calories. Summed scores of the 13 components yield a maximum score of 100, with a higher score reflecting greater compliance with the DGAs.

To devise a toddler-specific DQI, recommendations from the 2020–2025 DGAs for those 12 to 23.9 months of age were used, with a special focus on the foods and food amounts in the Healthy U.S.-Style Dietary Pattern for Toddlers Ages 12 Through 23 Months Who Are No Longer Receiving Human Milk or Infant Formula [9]. We also considered dietary recommendations from the Scientific Report of the 2020 Dietary Guidelines Advisory Committee, the American Academy of Pediatrics, the American Heart Association and selected National Academy of Medicine Dietary Reference Intake Reports [20,21].

The 2020–2025 DGAs focused on food components that maximize health benefits and included a range of nutrient and non-nutrient dietary constituents, such as macronutrients, micronutrients, fatty acids, and added sugars. In creating our index, we focused on toddlers ages 12 through 23 months. We excluded infants ages 6 to 11.9 months because they are just learning to eat complementary (table) foods and the variety and amounts consumed change substantially as the quantities and types are influenced by their consumption of breast milk and/or formula. Additionally, estimated average requirements have not been determined for most nutrients for children ages 6 to 11.9 months, whereas standards for nutrient adequacy exist for most nutrients for children ages 12 months and older [21]. Furthermore, as in the Healthy U.S.-Style Dietary Pattern for Toddlers, our index does not include children consuming human milk or infant formula. After 12 months infant formula is no longer recommended, and according to the most recent CDC Breastfeeding Report Card, most infants in the U.S. are no longer receiving breast milk (65%) [22]. Additionally, it is difficult to quantify the amount of breast milk consumed in this age group using common dietary assessment methods used in survey research, so survey methods rely on estimates [23,24]. Moreover, breast milk is highly variable in terms of its nutritional composition and the composition changes across time, with the concentrations of some nutrients varying based on maternal diet and the infant’s age [25].

The Scientific Report of the 2020 Dietary Guidelines Advisory Committee states that patterns of food group intake and category sources of food groups among those ages 12 to 23.9 months are similar to those ages 2 years and older [19]. This resulted in similar food- and nutrient-based recommendations for 12 to 23.9 month olds compared to those aged 2 years and older with some exceptions, which include avoiding added sugar entirely; consuming higher-fat dairy to ensure adequate intake of essential fatty acids and energy to support growth; prioritizing seafood; and making whole grains the predominant type of grains offered. Given the similarities in food patterns and the fact that dietary intake tracks across life stages, we leveraged the extensive work done to develop the HEI and used the HEI-2015 as a model for creating a diet quality index for toddlers ages 12 to 23.9 months (Table 1). However, as noted by the 2020 Dietary Guidelines Scientific Advisory Committee, toddlers have unique dietary needs for this rapid period of growth. Thus, to develop a scientifically accurate index that encouraged optimal health in this age group, we worked from the HEI-2015 model and informed our grading with empirical evidence from the 2020 Dietary Guidelines Scientific Advisory Committee and dietary recommendations issued by other expert bodies noted earlier, including the American Academy of Pediatrics and National Academy of Medicine Dietary Reference Intake Reports.

### 2.2. Diet Quality Index Components

Similar to the HEI, the components in the Toddler DQI include adequacy components, or food groups to encourage, and moderation components, or food groups to limit or decrease. The Toddler DQI consists of 14 components, 10 of which assess adequacy of the diet, including (1) whole fruit, (2) total vegetables, (3) greens and beans, (4) whole grains, (5) dairy, (6) total protein foods, (7) seafood, (8) plant proteins, (9) linoleic acid, and (10) alpha-linolenic acid. The remaining four assess dietary components recommended to consume in moderation: (11) 100% fruit juice, (12) refined grains, (13) sodium and (14) added sugars. Below, we note important deviations between the components in the HEI-2015 and our own index and the associated reasoning.

#### 2.2.1. 100% Fruit Juice

Unlike the HEI, we included 100% fruit juice as a moderation component because in 2019, the Academy of Pediatrics along with three other major health organizations produced a consensus statement recommending 100% juice be avoided for the first year of life and limited to no more than 4 ounces per day for 1 to 3 year olds, if given at all [26]. This report followed a policy statement from the Academy of Pediatrics in 2014 in which they note fruit juice offers no nutritional advantage over whole fruit as it has less fiber and can be consumed quicker, where, like sugar-sweetened beverages, it can contribute to an energy imbalance [27]. Consuming juice early in life may influence consumption of not only juice, but sugar-sweetened beverages later in life [28]. Thus, scoring for 100% fruit juice is not density based and is in accordance with Academy of Pediatrics recommendations; toddlers receive maximum points if they consume ≤4 oz of juice regardless of energy intake and minimum points if they consume >6 oz.

#### 2.2.2. Fats

Polyunsaturated fatty acids play a critical role in brain development during the first few years of life and evidence supports the need to provide foods that contain adequate amounts. The Dietary Guidelines Scientific Advisory Committee Report identified linoleic acid as a food component of concern given limited consumption among toddlers. Therefore, we included components to reflect adequate consumption of both linoleic and alpha-linolenic acids. We based standards for the maximum and minimum scores on the Dietary Reference Intakes Report developed and published by the Institute of Medicine (IOM). In the absence of sufficient evidence to develop a Recommended Daily Allowance, an adequate intake of 7 g/day for linoleic acid and 0.7 g/day for alpha-linolenic acid is recommended for toddlers. Unlike the HEI 2015, we did not include a ratio component of poly- and mono-unsaturated to saturated fatty acids or a moderation component for saturated fatty acid intake because the Scientific Advisory Committee Report noted that saturated fat intake is not of concern among this age group. In addition, saturated fat intake may be higher among toddlers given the recommendation to consume whole milk.

#### 2.2.3. Added Sugars

As explained in the Dietary Guidelines Scientific Advisory Committee Report, aiming to achieve recommended intakes of all key nutrients and food components leaves virtually no remaining energy for added sugars in the diets of toddlers. Therefore, the Toddler DQI awards maximum points for zero consumption of added sugar and no points when intake is 6% or more of total energy intake. For children this age, nutrient needs are high; but the amount toddlers can consume is low leaving little room for calories that contribute mostly energy rather than the nutrients necessary for optimal growth and development. Unfortunately, almost all children are exposed to added sugars by the time they are 2 years old [29]. This is concerning since added sugars can displace energy from nutrient-dense foods and increase the risk of nutrient inadequacies.

#### 2.2.4. Dairy

The standard for maximum points in the HEI-2015 for dairy is 1.3 cup equivalents per 1000 calories. Comparatively, the standard for maximum points in the Toddler DQI is 2.1 cup equivalents per 1000 calories. Milk is the largest contributor of energy among toddlers, which is appropriate for optimal growth and development. This is also to encompass shortfall nutrients, specifically calcium and vitamin D. Additionally, the dairy component for toddlers includes whole milk.

#### 2.2.5. Sodium

The sodium recommendations in the HEI-2015 were based on the recommended daily limit of 2300 mg in the 2015–2020 DGAs. At the time, the Tolerable Upper Intake Level (UL) for children aged 1 to 3 years was 1500 mg/day. In the 2020–2025 DGAs, the sodium guidelines were based on levels for Chronic Disease Risk Reduction (CDRR) defined by the National Academies of Sciences, Engineering, and Medicine in 2019 [20]. For children aged 1 to 3 years old, the 2019 recommendation is to limit sodium to 1200 mg/day. This CDRR for sodium was extrapolated from that for adults, which was established using evidence of the benefit of reducing sodium intake on cardiovascular and hypertension risk. Therefore, to achieve the maximum score for sodium in the Toddler DQI the target intake is ≤1200 mg/day. The minimum score is based on >1500 mg. We did not include a standard for having at least 800 mg of sodium, which is the updated level for adequate intake (AI) in the 2019 report, given the average intake of 12 to 23.9 month olds is 1586 mg per day and a reduction in sodium intake in lower calorie levels has been shown to be difficult to achieve [9,11].

### 2.3. Diet Quality Index Scoring

The HEI-2015 yields a maximum score of 100 based on the sum of components mapped to the 2015–2020 DGAs. The Toddler DQI also yields a maximum score of 100, but components are mapped to dietary recommendations outlined in the 2020–2025 DGAs (Table 1). For all components, higher scores reflect better diet quality such that for adequacy components, higher scores reflect higher intakes and for moderation components, which are reverse scored, higher scores reflect lower intakes because lower intakes are what is desired. A score of 100 corresponds to an optimal dietary pattern for toddlers, in accordance with recommendations from the revised DGAs and other expert bodies. Since total scores can be derived from a variety of eating patterns, it is important to also evaluate individual component scores to provide context and guidance for foods to consume more or less of while considering overall energy intake.

Thresholds for the maximum and minimum scoring standards were determined using the least restrictive recommendations among the 800–1000 calorie range from the Healthy U.S.-Style Dietary Pattern for Toddlers in the 2020–2025 DGAs, as this is the estimated calorie range for 12 to 23.9 months olds [9]. However, unlike the HEI, which is meant for a range of ages and populations, not all components of the Toddler DQI are density based (e.g., per 1000 calories). The dietary recommendations for toddlers at the 800–1000 calorie level include consuming 2.0 ounce equivalents (oz-eq) per day of both whole grains and total protein and 2.0 oz-eq per week of seafood. This reflects the need for adequate intake of nutrient-rich foods. Additionally, as mentioned above, this is when eating habits and food preferences form, impacting diet-related outcomes across the life course. Thus, exposing children to a variety of foods, including seafood, is important during this life stage. Therefore, toddlers receive the maximum scores for the protein foods, whole grains, and seafood index components if they consume 2.0 oz-eq per day of protein foods, 2 oz-eq per day of whole grains, and 2.0 oz-eq per week of seafood. Except for sodium and 100% fruit juice, all other components rely on densities (amount per 1000 kcals), with scores assigned to each component by comparing the density to the relevant standards. Minimum and maximum scoring standards are shown in Table 1. For all components, densities between the minimum and maximum standards are scored proportionately.

Similar to the HEI-2015 we applied a weighting system to all components. We decided that some food categories should carry more weight due to the life stage of toddlers and the importance of solidifying taste preference (see Table 1). For example, the vegetables (total vegetables and greens and beans combined) and fruits (whole fruits and 100% fruit juice combined) are weighted to receive a maximum of 15 points each. These food categories were given greater weight because providing frequent exposure to a variety of vegetables and fruit is critically important among this age group, for it shapes future eating behaviors and preferences. Eating fewer vegetables and fruit during infancy sets children up to eat less of these when they are older [5]. In addition, according to data from What We Eat in America, NHANES 2007–2016, approximately 90% of toddlers are falling short of recommended daily intakes for vegetables and approximately 40% of toddlers do not meet recommended intakes for fruit, with most coming from 100% fruit juice [9].

### 2.4. Sampling and Data Collection

The FITS 2016 was a nationally distributed, cross-sectional survey. The details for sampling selection and procedures are described elsewhere [30]. Following administration of a household survey by trained interviewers, dietary intake data were collected over the telephone from parents or caregivers of young children. Respondents were mailed materials to assist them with accurate reporting of portion sizes for their 24 h dietary recall. These included a ruler, a measuring cup, and a Food Measurement Aids booklet. All materials were in both English and Spanish. Respondents were asked to measure cups used by the child to drink beverages (e.g., sippy cups) per the provided instructions before the recall. The 24 h recalls were completed by telephone by trained and certified dietary interviewers from the Nutrition Coordinating Center (University of Minnesota, Minneapolis, MN) using the Nutrient Data System for Research (NDSR) 2015. A second 24 h recall was conducted on 25% of the respondents who completed a first 24 h recall, at least one week later. Respondents for the second 24 h recall were selected at random during the recruitment phase. Informed consent was obtained from all participants involved in the study.2.5. Statistical Methods

For the analyses reported in this paper, the analytic sample was limited to those aged 12 to 23.9 months who did not consume any breast milk or formula (*n* = 882). We aimed to describe average total and component scores and compare average scores by group. Thus, for those with two days of dietary intake data, only the first day of intake was used for analyses. Sampling weights were calculated to account for the probability of household selection and then adjusted for nonresponse and incomplete coverage to be nationally reflective of the U.S. population aged birth to 47.9 months. Toddler sociodemographic characteristics are summarized as frequencies (%) for categorical variables and means and standard errors (SE) for continuous variables. Toddler DQI scores were calculated using NDSR output. Each component was estimated using methods outlined by the Nutrition Coordinating Center for the HEI-2015 using 1 recall day for each participant (http://www.ncc.umn.edu/healthy-eating-index-hei/, accessed 17 December 2020). Total Toddler DQI and Toddler DQI component scores are summarized as means and SE. The percent of maximum score achieved [(mean score/maximum score) × 100%], is also presented since maximum scores differed for various components and ranged from 2.5 to 10. Covariates included sociodemographic variables thought to influence diet quality, including child race and ethnicity; first born; WIC eligibility status, as either self-reported or calculated based on income and household size; an indicator for a parent or caregiver reporting no other adults living in their household; a continuous measure of federal poverty level index (FPL); and urban/rural location of the child’s household. FPL index is defined as a percentage at which the household income is compared to the poverty line, meaning 100% would mean that a given household is at the exact federal poverty line and anything over would be above poverty. Non-Hispanic other race children estimates are not presented due to insufficient sample sizes in the data, although they were included in the analysis. Wald F-tests were conducted to determine statistical significance within each covariate for overall Toddler DQI and each food component. If the overall test was statistically significant at the 0.05 level, pairwise t-tests of the least square means were conducted. Analyses are presented as adjusted for ever breastfed, WIC participation status, race and ethnicity, federal poverty level, households with one adult, and living in urban settings. Multivariate regression was conducted for each Toddler DQI component. All statistical analyses were performed with SAS (version 9, SAS Institute Inc., Cary, NC, USA) and SAS-callable SUDAAN^®^ (version 11, RTI International, Research Triangle Park, NC, USA) software.

## 3. Results

### 3.1. Sample Characteristics

In our unweighted sample, most children had been breastfed (77.1%), approximately one-third were the first born (33.9%), and half were male (50.8%) (Table 2). The majority of children were non-Hispanic white (65.9%), 15.1% percent were non-Hispanic Black, 15.0% were Hispanic, and 4.0% were non-Hispanic other race. Approximately half of the sample had a household income less than $50,000/year (52.5%), 32.7% had a household income between $50,000 and $100,000/year and 14.9% had a household income greater than $100,000/year. Approximately half of the caregivers in our sample (53.9%) had a college education or more. Approximately one-third of children participated in WIC (35.1%). Finally, most children lived in a household with more than one adult (92.1%) and in urban settings (80.3%).

### 3.2. Overall Toddler DQI and Components Scores

The mean Toddler DQI score was 48.6 (0.6), out of 100 maximum points (Table 3). The mean scores for total vegetables and greens and beans were 4.3 (0.1) out of a maximum of 10 points and 1.1 (0.1) out of a maximum of 5 points, respectively. The mean score for whole fruit was 6.2 (0.2) out of 10 points. The mean score for 100% fruit juice was 3.4 (0.1) out of 5 points. For whole grain intake, the mean component score was 3.8 (0.2) out of 10 points. The mean dairy score was 7.9 (0.1) out of 10 points. The mean score for total protein intake was 3.8 (0.1) out of 5 points. Mean scores for seafood and plant protein were 0.2 (0.0) and 0.8 (0.1) out of a maximum of 2.5 points, respectively. The mean score for refined grains was 2.4 (0.2) and the mean score for added sugars was 2.3 (0.1) out of a maximum of 10 points. For sodium, the mean score was 4.4 (0.2) out of 10 points. Finally, the mean scores for intake of the unsaturated fatty acids linolenic and alpha linoleic were 3.6 (0.1) and 4.4 (0.0) both out of a maximum of 5 points, respectively.

**Figure 1:**
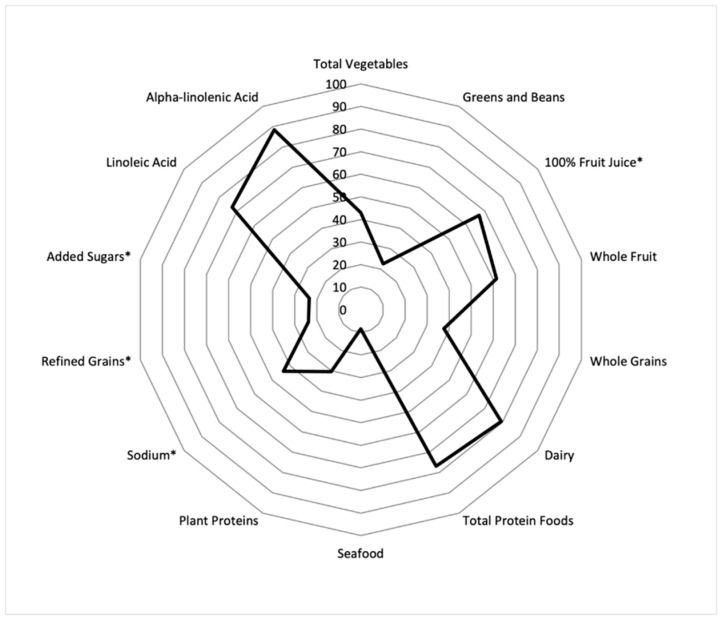
Radar plot showing the percent of the maximum score achieved for each of the Toddler Diet Quality Index components among toddlers 12 to 23.9 months old in the Feeding Infants and Toddler Study 2016 (*n* = 882). * Moderation components—higher score indicates lower consumption.

The radar plot in Figure 1 shows the percent of the maximum score achieved for each component simultaneously; a perfect Toddler DQI total score (100% for each component) would be displayed as a line around the border of the radar plot. As demonstrated, toddlers are consuming close to adequate amounts of alpha-linolenic acid (88% of the maximum score). Others that were close to the maximum score included dairy (79%), total protein foods (77%), linoleic acid (73%), and fruit juice (67%). Components that were furthest from the maximum score included seafood (8%), greens and beans (23%), added sugars (23%), refined grains (24%), and plant proteins (30%). The remaining components included whole grains (38%), total vegetables (43%), sodium (44%), and whole fruit (62%).

### 3.3. Demographic Differences in DQI Scores

#### 3.3.1. Breastfeeding Behavior

In adjusted analyses, toddlers in our sample that were never breastfed had lower total Toddler DQI scores (45.9) than toddlers that were ever breastfed (49.5) (Table 3). Similarly, toddlers that were never breastfed also had lower component scores for greens and beans and whole grains (0.8 and 2.9, respectively) than toddlers who were ever breastfed (1.3 and 4.0). There were no significant differences in other component scores by prior breastfeeding behavior.

#### 3.3.2. Race and Ethnicity

Hispanic toddlers in our sample had the highest overall adjusted mean Toddler DQI score (51.8) followed by non-Hispanic white toddlers (47.4) and non-Hispanic Black toddlers (46.5). Non-Hispanic Black toddlers had the highest mean component score for vegetables (5.1) followed by Hispanic toddlers (4.5) and non-Hispanic white toddlers (3.9). Hispanic and non-Hispanic Black toddlers had the same component score for greens and beans (1.4) and non-Hispanic white toddlers had a lower greens and beans score (0.9). Non-Hispanic Black toddlers had a lower mean component score for dairy (7.1) compared to Hispanic (8.0) and non-Hispanic white toddlers (8.1). Hispanic toddlers had the highest mean component scores for sodium, added sugars, and refined grains (5.5, 3.2, and 3.4, respectively) followed by non-Hispanic white toddlers (3.8, 2.1, and 2.0, respectively) and non-Hispanic Black toddlers (3.5, 2.1, and 1.4, respectively). There were no significant differences by race or ethnicity in component scores for 100% fruit juice, whole fruit, total protein foods, seafood, plant proteins, whole grains, linolenic acid, or alpha linoleic acid.

#### 3.3.3. WIC Participation

In our sample, toddlers who were not eligible for WIC based on income adjusted for household size had the highest overall mean adjusted Toddler DQI score (51.2), followed by toddlers who were income eligible but not participating in WIC (48.7) and WIC participants (46.6). Income-ineligible toddlers also had higher component scores for total vegetables and 100% fruit juice (4.8 and 3.8, respectively) than income-eligible non-participants (4.6 and 3.4, respectively) and WIC participants (3.9 and 3.0, respectively). Income-eligible non-WIC participants had the highest component scores for whole fruit (6.8) followed by income-ineligible toddlers (6.7) and WIC participants (5.6). There were no significant differences by WIC participation status in mean component scores for greens and beans, whole grains, total protein foods, seafood, plant proteins, sodium, refined grains, added sugar, linolenic acid, or alpha linoleic acid.

#### 3.3.4. Federal Poverty Level (FPL) Index

Adjusted mean overall Toddler DQI scores increased by 0.7 points per 100-point increase in the FPL index in our sample, meaning toddlers living in higher-income households had higher mean DQI scores. Similarly, toddlers in higher-income households had higher component scores for 100% juice (increase of 0.1 points per 100-point increase in FPL index), meaning they drank less. There were no significant differences in any of the other adjusted mean component scores by household FPL index.

#### 3.3.5. Urbanicity

There was no significant difference in the overall mean adjusted Toddler DQI scores between toddlers living in urban (49.1) and rural households (46.5). Toddlers living in urban households had higher mean component scores for greens and beans (1.2) than toddlers living in rural households (0.8). All other differences in adjusted mean component scores were not significant.

## 4. Discussion

The updated 2020–2025 DGAs include for the first time dietary recommendations for infants and toddlers. Eating in alignment with the DGAs is most often assessed using the HEI [31], a validated measure of diet quality. However, the existing HEI was designed for ages 2 and over and is not appropriate for children younger than 2 years old. Therefore, we developed a new diet quality index for those 12 to 23.9 months of age who are no longer consuming breast milk or infant formula. This new index, called the Toddler DQI, was modeled after the most recent version of the HEI, the HEI-2015 using recommendations from the 2020–2025 DGAs and other expert bodies. Using data from the FITS 2016 survey, preliminary construct testing was carried out to see if this new index functions as expected. Using a national sample of toddlers, our results show the diet quality of toddlers aged 12 to 23.9 months is poor; with a score of 49 out of 100, indicating that there is vast room for improvement. Toddlers consumed too little seafood, greens and beans, and plant proteins and too many refined grains and added sugars. Our results found that Toddler DQI scores were higher among children who were ever breastfed, lived in households with higher incomes, and who were Hispanic.

Given the lack of comprehensive dietary guidelines for infants and toddlers in the U.S., few have assessed diet quality among young children, making it difficult to compare our results against scoring norms. Hamner and Moore modified the Diet Quality Index Score (DQIS) and used NHANES data to assess diet quality in children 6 months to 4 years of age [18]. Although standards were based on alternative sources of information, such as the Child and Adult Care Food Program, some aspects of the DQIS are similar to the Toddler DQI, such as allotting maximum points for zero consumption of added sugars and consuming <4.0 oz/day of 100% fruit juice. However, the Toddler DQI allots maximum points for ≥2.0 oz-eq of whole grains, something known to be lacking in the diets of children, where the DQIS allots minimum points to children who have 0 or >2.0 oz-eq/day. Nevertheless, it is valuable to compare scores from each index to get an idea of how children in the U.S. are eating. Using the DQIS, 1 year olds scored 53% of maximum points, which is only marginally higher than our average score of 49% of maximum points.

Au et al. assessed diet quality among 13 month olds and 24 month olds enrolled in WIC using the HEI-2015; the average HEI score for both groups was 64 (out of 100) [15]. However, as noted in the 2015–2020 DGAs, the HEI-2015 is not designed for children under 2 years of age [8]. Compared to the Toddler DQI, the HEI-2015 allows for added sugar, more refined grains and less whole grains for maximum scoring for these index components. Given the high nutrient needs and small stomach capacity of young children, the standards in the HEI-2015 are not meant to be extrapolated to infants and toddlers, who require specific portion sizes appropriate for their age and development [32]. In fact, a large emphasis of the 2020–2025 DGAs includes shifting from the notion that “food under one is just for fun” to “every bite counts”, highlighting that young children have very little, if any, discretionary calorie allotments that enable consumption of added sugars and other nutrients and food groups in excess of recommended intake the way older children and adults do [9]. Further, the maximum and minimum scoring standards for the HEI-2015 encompass a range of calories from 1200 to 2400 kcal, a range vastly different from the 800–1000 kcal most toddlers consume on a daily basis [12]. Thus, it is not surprising that the HEI-2015 score among this age group in the Au et al. study was significantly higher than the Toddler DQI in the present study.

Prior studies have shown that diet quality is often better among children receiving WIC benefits compared to children of similar economic status not receiving WIC [33,34,35,36]. This may explain higher diet quality scores in Au et al., since the sample included children from the WIC Infant and Toddler Feeding Practices Study 2 (WIC ITFPS-2), where 86% of 13 month olds and 69% of 24 month olds were still enrolled in WIC at the time of data collection. This is compared to the 35% enrolled in WIC in our sample. A study by Weinfield et al. also included children from the WIC ITFPS-2 but included only those with long-term follow up through 24 months of life [37]. In that study, 82% had continued participation in WIC through most of the first 2 years of life. The mean adjusted HEI-2015 was 60.4 and those who were in WIC longer had higher HEI scores. However, in our study, toddlers who were receiving WIC benefits had lower diet quality compared to their non-WIC counterparts. A study by Guthrie et al. using FITS 2016 data demonstrated poorer dietary habits among 12 to 23.9 month olds receiving WIC, including consuming more SSBs and fruit juice and less fruit compared to their non-WIC counterparts [38]. As mentioned above, the Toddler DQI does not allow for energy dense, nutrient-poor foods, per the 2020–2025 DGAs, and applies greater weight to fruits and vegetables compared to the HEI-2015. Prior to the COVID-19 pandemic, the maximum monthly allowance WIC participants could receive for fruits and vegetables was $11 in cash value vouchers and up to 144 fl oz in juice [39]. Therefore, it is not surprising that WIC participants in our study had significantly lower scores for whole fruit, total vegetables and 100% fruit juice. As stated in a 2017 report by the National Academy of Sciences, the WIC food packages can be updated to better align with the DGAs; they specifically suggest increasing the dollar amount of the fruit and vegetable cash value voucher and reducing the amounts of juice in all food packages to improve the balance of food groups in alignment with the 2015–2020 DGAs [40]. Thus, although the WIC food packages provide nutrient-rich foods to supplement the diets of young children, it may not be enough to have significant impacts on toddler diet quality.

Similar to results from other studies, young children’s diets are lacking in seafood [41]. In the 2017 National Academy of Sciences report, the committee recommended adding seafood to the WIC food packages given evidence of limited consumption. Few children in the FITS 2016 data set ate seafood, as demonstrated by the extremely low scores (8% of the maximum score). Thus, the emphasis on seafood in the 2020–2025 DGAs and potential inclusion in the WIC food package could benefit diet quality, but strategies to encourage acceptance may be needed. Fatty fish, such as salmon and tuna, are among the few foods that contain high enough levels of essential fatty acids in a portion size appropriate for very young children, but they are not often offered due to concerns about food allergies or mercury content [42]. Before we recommend increased intake of seafood, it is important to address concerns and clarify the benefits that consuming seafood can have on children’s physical and brain development.

Our results show differences in diet quality by race and ethnicity even among this young age group. Other studies demonstrate differences in diet based on origin of race or ethnicity in young children. Hamner et al. found differences as early as 6 months old [43]. Among non-Hispanic Black, Hispanic and non-Hispanic white 1 year olds, they found that more non-Hispanic Black children had 100% fruit juice, while fewer non-Hispanic white children had SSBs compared to their counterparts from other races and ethnicities. Fewer Hispanic 1 year olds had grains and snacks, which aligns with our results of Hispanic toddlers having less refined grains and sodium, often found in snacks, compared to non-Hispanic Black and non-Hispanic white children. Davis et al. found differences in sodium levels, with Mexican Americans consuming less than non-Hispanic Black and non-Hispanic white 0 to 24 month olds [44]. Traditional Hispanic diets often include an emphasis on vegetables, legumes, and whole grains, thus offering a potential explanation for the higher diet quality scores found among Hispanic toddlers [45,46]. This presents an opportunity to offer targeted interventions promoting the use of legumes and beans that are often found in traditional Hispanic and Latino dishes, to increase diet quality.

Our study had a few limitations. We used data from a single 24 h recall; describing diet quality on a given day does not necessarily capture usual intake of a child. Toddler dietary intake was reported by the parent or caregiver, which could lead to over- or underestimation of overall intake. To avoid incomplete dietary recalls, FITS uses a form sent to childcare providers to collect information about foods fed by other adults. We excluded children receiving breast milk or formula given the inability of the Dietary Guidelines Scientific Advisory Committee to provide a recommended food pattern due to uncertainty about the associated nutrient intakes. However, in sensitivity analyses including children receiving breast milk one time per day, results were not significantly different. Although we adjusted for sociodemographic characteristics known to influence feeding behaviors, there is the possibility of residual confounding due to measurement error or selection of covariate categories. It is important to note that racial and ethnic categories are social constructs rather than biological designations and differences in intakes and feeding practices according to race, ethnicity, and income are correlated and impossible to fully disentangle. Lastly, the large number of comparisons performed can lead to inflated type 1 error rates. However, the focus of this study was to report construct validity, not present statistical comparisons. A strength of our study is the use of a large sample weighted to be nationally representative, allowing us to assess differences in diet quality by a range of sociodemographic characteristics. We adapted an existing food coding scheme for categories from the USDA’s Food Patterns Equivalent Database. Lastly, this is the first study to develop a Toddler DQI that is modeled after the HEI; we based our scoring determination on existing recommendations from the most recent DGAs, the American Academy of Pediatrics, and the American Heart Association.

## 5. Conclusions

Our results support the 2020–2025 DGAs in that “every bite counts” and toddlers need more opportunities to consume nutrient-rich foods in place of energy dense, nutrient-poor foods. Our findings are largely in line with what has been reported in the literature using other diet quality indices. However, this is the first study to report on diet quality among a nationally distributed group of toddlers using federal nutrition guidelines, as the DGAs included toddlers in their recommendations for the first time. The Toddler Diet Quality Index could be adapted for use in other in countries with dietary guidance that is similar to that in the U.S. Future research can include assessing the validity of the Toddler DQI in other populations. Interventions should focus on supporting parents and caregivers in modeling healthy eating behaviors and involve children in food purchasing and preparation to foster positive eating habits they will maintain into adulthood. Findings from this study support focusing nutrition education messages on increasing intake of fruits, vegetables, legumes, whole grains, and seafood, while reducing consumption of added sugars. During this formative period, it is important to increase exposure to essential fatty acids and decrease exposure to foods with added sugar and sodium. Dietary habits among US toddlers need to be improved because habits developed early influence dietary preferences for life.

## Figures and Tables

**Table 1:** Comparison of components and scoring standards for the Toddler Diet Quality Index for U.S. children aged 12 to 23.9 months not consuming human milk or infant formula and the Healthy Eating Index 2015 for those aged 2 years and older in the U.S.

	Toddler Diet Quality Index			Healthy Eating Index 2015		
Component	Max Points	Standard for Max Score	Standard for Min Score	Max Points	Standard for Max Score	Standard for Min Score
Food and Nutrients to Increase						
Total Fruits ^1^		NA	NA	5	≥0.8 c eq per 1000 kcal	No fruit
Whole Fruits ^2^	10	≥0.48 c eq per 1000 kcal	No whole fruit	5	≥0.4 c eq per 1000 kcal	No whole fruit
Total Vegetables	10	≥0.95 c eq per 1000 kcal	No vegetables	5	≥1.1 c eq per 1000 kcal	No vegetables
Greens and Beans	5	≥0.12 c eq per 1000 kcal	No dark green vegetables or legumes	5	≥0.2 c eq per 1000 kcal	No dark green vegetables or legumes
Whole Grains	10	≥2.0 oz eq/day	No whole grains	10	≥1.5 oz eq per 1000 kcal	No whole grains
Dairy ^3^	10	≥2.1 oz eq per 1000 kcal	No dairy	10	≥1.3 c eq per 1000 kcal	No dairy
Total Protein Foods	5	≥2.0 oz eq/day	No protein foods	5	≥2.5 oz eq per 1000 kcal	No protein foods
Seafood and Plant Proteins ^4^		NA	NA	5	≥0.8 c eq per 1000 kcal	No seafood or plant proteins
Seafood	2.5	≥0.29 c eq/day	No seafood		NA	NA
Plant Proteins	2.5	≥0.25 c eq per 1000 kcal	No plant proteins or legumes		NA	NA
Linoleic Acid	5	≥6.3% of energy	No linoleic acid		NA	NA
Alpha Linolenic Acid	5	≥0.63% of energy	No alpha linolenic acid		NA	NA
Fatty Acids ^5^		NA	NA	10	(PUFAs+MUFAs)/SFAs ≥2.5	(PUFAs+MUFAs)/SFAs ≤1.2
Food and Nutrients to Limit or Decrease						
100% Fruit Juice	5	≤4 oz/day	≥6 oz/day		NA	NA
Refined Grains	10	≤0.6 oz eq per 1000 kcal	≥2.0 oz eq per 1000 kcal	10	≤1.8 oz eq per 1000 kcal	≥4.3 oz eq per 1000 kcal
Sodium	10	≤1.2 g/day	≥1.5 g/day	10	≤1.1 g per 1000 kcal	≥2.0 g per 1000 kcal
Added Sugars	10	No added sugars	≥6% of energy	10	≤6.5% of energy	≥26% of energy
Saturated Fats				10	≤8% of energy	≥16% of energy

^1^ Includes100% fruit juice. ^2^ Includes all forms except juice. ^3^ Includes all milk products, such as fluid milk, yogurt, and cheese, and fortified soy beverages. ^4^ Includes seafood, nuts, seeds, soy products (other than beverages), and legumes (beans and peas). ^5^ Ratio of poly- and mono-unsaturated fatty acids (PUFAs and MUFAs) to saturated fatty acids (SFAs).

**Table 2:** Unweighted distributions and selected estimates (percent and standard error) among children aged 12–23.9 months not receiving breast milk or formula from the Feeding Infants and Toddlers Study 2016 (*n* = 882).

Characteristic		%	SE
Male		50.8	1.7
Ever Breastfed		77.1	1.4
Child First Born		33.9	1.8
Child race/ethnicity	Hispanic	15.0	1.2
	Non-Hispanic white	65.9	1.6
	Non-Hispanic Black	15.1	1.2
	Non-Hispanic Other	4.0	0.7
Household income (in Dollars)	Under 10,000	9.2	1.0
	10,000 to 19,999	8.5	0.9
	20,000 to 34,999	17.9	1.3
	35,000 to 49,999	16.9	1.3
	50,000 to 74,999	20.1	1.4
	75,000 to 99,999	12.6	1.1
	100,000 to 149,999	10.7	1.0
	150,000 or more	4.2	0.7
Maternal education	High school or less	22.6	1.4
	Some post-secondary	23.5	1.4
	College or graduate work	53.9	1.7
WIC Participant		35.1	1.6
Urbanicity	Urban	80.3	1.3
	Rural	19.7	1.3

**Table 3:** Toddler Diet Quality Index (DQI) total and component scores (mean and standard error) for children 12 to 23.9 months old from the Feeding Infants and Toddlers Study 2016 and adjusted analyses for selected sociodemographic characteristics ^1^.

Children 12–23.9 Months (*n* = 882)		Ever Breastfed		Race/ Ethnicity			WIC			Poverty Level
		No (*n* = 200)	Yes (*n* = 675)	Hispanic (*n* = 132)	Non-Hispanic White (*n* = 577)	Non-Hispanic Black (*n* = 131)	WIC (*n* = 306)	WIC Eligible (*n* = 160)	Non-WIC (*n* = 409)	Per 100% Increase (*n* = 875)
Total Toddler DQI	48.6 (0.6)	45.9 (1.0)	49.5 (0.6)	51.8 (1.1)	47.4 (0.6)	46.5 (1.4)	46.6 (0.9)	48.7 (1.1)	51.2 (1.0)	0.7 (0.3)
Foods and Nutrients to Increase										
Total vegetable	4.3 (0.1)	4.0 (0.3)	4.4 (0.2)	4.5 (0.4)	3.9 (0.2)	5.1 (0.4)	3.9 (0.2)	4.6 (0.3)	4.8 (0.3)	−0.1 (0.1)
Greens and beans	1.1 (0.1)	0.8 (0.2)	1.3 (0.1)	1.4 (0.2)	0.9 (0.1)	1.4 (0.2)	0.9 (0.1)	1.3 (0.2)	1.4 (0.2)	0.0 (0.1)
Whole fruit	6.2 (0.2)	5.5 (0.4)	6.4 (0.2)	6.1 (0.4)	6.4 (0.2)	5.8 (0.5)	5.6 (0.3)	6.8 (0.4)	6.7 (0.3)	0.2 (0.1)
Whole grains	3.8 (0.2)	2.9 (0.3)	4.0 (0.2)	3.7 (0.3)	3.8 (0.2)	3.6 (0.4)	3.7 (0.3)	3.6 (0.3)	3.9 (0.3)	0.1 (0.1)
Dairy	7.9 (0.1)	8.0 (0.2)	7.9 (0.1)	8.0 (0.3)	8.1 (0.1)	7.1 (0.3)	7.9 (0.2)	7.7 (0.3)	8.2 (0.2)	0.1 (0.1)
Total proteins	3.8 (0.1)	3.8 (0.1)	3.9 (0.1)	3.8 (0.2)	3.9 (0.1)	4.1 (0.2)	3.9 (0.1)	4.0 (0.2)	3.7 (0.1)	0.0 (0.1)
Seafood	0.2 (0.0)	0.1 (0.1)	0.2 (0.0)	0.1 (0.1)	0.2 (0.0)	0.3 (0.1)	0.2 (0.1)	0.2 (0.1)	0.2 (0.1)	0.0 (0.0)
Plant proteins	0.8 (0.1)	0.7 (0.1)	0.8 (0.1)	0.8 (0.1)	0.8 (0.1)	0.9 (0.1)	0.8 (0.1)	0.7 (0.1)	0.8 (0.1)	0.1 (0.0)
Linoleic acid	3.6 (0.1)	3.8 (0.1)	3.6 (0.1)	3.6 (0.1)	3.7 (0.1)	3.7 (0.1)	3.6 (0.1)	3.8 (0.1)	3.6 (0.1)	−0.1 (0.0)
Alpha-linolenic acid	4.4 (0.0)	4.5 (0.1)	4.4 (0.1)	4.5 (0.1)	4.4 (0.1)	4.4 (0.1)	4.4 (0.1)	4.4 (0.1)	4.4 (0.1)	0.0 (0.0)
Foods and Nutrients to Limit or Decrease										
100% fruit juice	3.4 (0.1)	3.1 (0.2)	3.4 (0.1)	3.2 (0.2)	3.5 (0.1)	3.3 (0.3)	3.0 (0.2)	3.4 (0.2)	3.8 (0.2)	0.1 (0.1)
Sodium	4.4 (0.2)	4.7 (0.4)	4.3 (0.2)	5.5 (0.5)	3.8 (0.2)	3.5 (0.5)	4.2 (0.3)	3.7 (0.4)	5.0 (0.4)	0.2 (0.1)
Refined grains	2.4 (0.2)	1.9 (0.3)	2.5 (0.2)	3.2 (0.4)	2.1 (0.2)	2.1 (0.4)	2.2 (0.3)	2.5 (0.3)	2.6 (0.3)	0.1 (0.1)
Added sugars	2.3 (0.1)	2.2 (0.3)	2.4 (0.1)	3.4 (0.4)	2.0 (0.1)	1.4 (0.3)	2.4 (0.2)	2.2 (0.3)	2.3 (0.2)	0.1 (0.1)

^1^ Adjusted for ever breastfed, WIC participation status, race and ethnicity, federal poverty level, households with one adult and living in urban settings; data not shown for households with one adult and living in urban settings. Shaded results indicate significant difference for the group, *p* < 0.05.

## References

[1] Golley R.K., Smithers L.G., Mittinty M.N., Emmett P., Northstone K., Lynch J.W. (2013). Diet quality of U.K. infants is associated with dietary, adiposity, cardiovascular, and cognitive outcomes measured at 7–8 years of age. J. Nutr..

[2] McGuire S. (2012). Institute of Medicine (IOM) Early Childhood Obesity Prevention Policies. Washington, DC: The National Academies Press; 2011. Adv. Nutr..

[3] Miles G., Siega-Riz A.M. (2017). Trends in Food and Beverage Consumption among Infants and Toddlers: 2005–2012. Pediatrics.

[4] Birch L.L., Doub A.E. (2014). Learning to eat: Birth to age 2 y. Am. J. Clin. Nutr..

[5] Grimm K.A., Kim S.A., Yaroch A.L., Scanlon K.S. (2014). Fruit and vegetable intake during infancy and early childhood. Pediatrics.

[6] Park S., Pan L., Sherry B., Li R. (2014). The association of sugar-sweetened beverage intake during infancy with sugar-sweetened beverage intake at 6 years of age. Pediatrics.

[7] Rose C.M., Birch L.L., Savage J.S. (2017). Dietary patterns in infancy are associated with child diet and weight outcomes at 6 years. Int. J. Obes..

[8] U.S. Department of Health and Human Services and U.S. Department of Agriculture (2015). 2015–2020 Dietary Guidelines for Americans. 8th Edition. https://health.gov/our-work/food-nutrition/previous-dietary-guidelines/2015.

[9] U.S. Department of Agriculture and U.S. Department of Health and Human Services (2020). Dietary Guidelines for Americans, 2020–2025. 9th Edition. DietaryGuidelines.gov.

[10] Kennedy E.T., Ohls J., Carlson S., Fleming K. (1995). The Healthy Eating Index: Design and applications. J. Am. Diet. Assoc..

[11] Reedy J., Lerman J.L., Krebs-Smith S.M., Kirkpatrick S.I., Pannucci T.E., Wilson M.M., Subar A.F., Kahle L.L., Tooze J.A. (2018). Evaluation of the Healthy Eating Index-2015. J. Acad. Nutr. Diet..

[12] Krebs-Smith S.M., Pannucci T.E., Subar A.F., Kirkpatrick S.I., Lerman J.L., Tooze J.A., Wilson M.M., Reedy J. (2018). Update of the Healthy Eating Index: HEI-2015. J. Acad. Nutr. Diet..

[13] Thomson J.L., Tussing-Humphreys L.M., Goodman M., Landry A.S. (2019). Diet quality in a nationally representative sample of American children by sociodemographic characteristics. Am. J. Clin. Nutr..

[14] Dalwood P., Marshall S., Burrows T.L., McIntosh A., Collins C.E. (2020). Diet quality indices and their associations with health-related outcomes in children and adolescents: An updated systematic review. Nutr. J..

[15] Au L.E., Gurzo K., Paolicelli C., Whaley S.E., Weinfield N.S., Ritchie L.D. (2018). Diet Quality of US Infants and Toddlers 7–24 Months Old in the WIC Infant and Toddler Feeding Practices Study-2. J. Nutr..

[16] Marshall S., Burrows T., Collins C.E. (2014). Systematic review of diet quality indices and their associations with health-related outcomes in children and adolescents. J. Hum. Nutr. Diet..

[17] Kourlaba G., Panagiotakos D.B. (2009). Dietary quality indices and human health: A review. Maturitas.

[18] Hamner H.C., Moore L.V. (2020). Dietary quality among children from 6 months to 4 years, NHANES 2011-2016. Am. J. Clin. Nutr..

[19] Dietary Guidelines Advisory Committee (2020). Scientific Report of the 2020 Dietary Guidelines Advisory Committee: Advisory Report to the Secretary of Agriculture and the Secretary of Health and Human Services.

[20] Stallings V.A., Harrison M., Oria M., National Academies of Sciences, Engineering, and Medicine (2019). Dietary Reference Intakes for Sodium and Potassium.

[21] Otten J.J., Hellwig J.P., Meyers L.D., Institute of Medicine (2006). Dietary Reference Intakes: The Essential Guide to Nutrient Requirements.

[22] Centers for Disease Control and Prevention (2020). Breastfeeding Report Card United States, 2020. https://www.cdc.gov/breastfeeding/pdf/2020-Breastfeeding-Report-Card-H.pdf.

[23] Ahluwalia N., Herrick K.A., Rossen L.M., Rhodes D., Kit B., Moshfegh A., Dodd K.W. (2016). Usual nutrient intakes of US infants and toddlers generally meet or exceed Dietary Reference Intakes: Findings from NHANES 2009–2012. Am. J. Clin. Nutr..

[24] Bailey R.L., Catellier D.J., Jun S., Dwyer J.T., Jacquier E.F., Anater A.S., Eldridge A. (2018). Total Usual Nutrient Intakes of US Children (Under 48 Months): Findings from the Feeding Infants and Toddlers Study (FITS) 2016. J. Nutr..

[25] Spahn J.M., Callahan E.H., Spill M.K., Wong Y.P., Benjamin-Neelon S.E., Birch L., Black M.M., Cook J.T., Faith M.S., Mennella J. (2019). Influence of maternal diet on flavor transfer to amniotic fluid and breast milk and children’s responses: A systematic review. Am. J. Clin. Nutr..

[26] Lott M., Callahan E., Duffy E.W., Story M., Daniels S. (2019). Healthy Beverage Consumption in Early Childhood: Recommendations from Key National Health And Nutrition Organizations.

[27] Heyman M.B., Abrams S.A. (2017). Fruit Juice in Infants, Children, and Adolescents: Current Recommendations. Pediatrics.

[28] Sonneville K.R., Long M., Rifas-Shiman S.L., Kleinman K., Gillman M.W., Taveras E.M. (2015). Juice and water intake in infancy and later beverage intake and adiposity: Could juice be a gateway drink?. Obesity.

[29] Herrick K.A., Fryar C.D., Hamner H.C., Park S., Ogden C.L. (2020). Added Sugars Intake among US Infants and Toddlers. J. Acad. Nutr. Diet..

[30] Anater A.S., Catellier D.J., Levine B.A., Krotki K.P., Jacquier E.F., Eldridge A., Bronstein K.E., Harnack L.J., Peasley J.M.L., Lutes A.C. (2018). The Feeding Infants and Toddlers Study (FITS) 2016: Study Design and Methods. J. Nutr..

[31] Suthutvoravut U., Abiodun P.O., Chomtho S., Chongviriyaphan N., Cruchet S., Davies P.S.W., Fuchs G.J., Gopalan S., van Goudoever J.B., Nel E.R. (2015). Composition of Follow-Up Formula for Young Children Aged 12–36 Months: Recommendations of an International Expert Group Coordinated by the Nutrition Association of Thailand and the Early Nutrition Academy. Ann. Nutr. Metab..

[32] Pérez-Escamilla R., Segura-Pérez S., Lott M. (2017). Feeding Guidelines for Infants and Young Toddlers: A Responsive Parenting Approach. Nutr. Today.

[33] Tester J.M., Leung C.W., Crawford P.B. (2016). Revised WIC Food Package and Children’s Diet Quality. Pediatrics.

[34] Andreyeva T., Luedicke J., Henderson K.E., Schwartz M.B. (2014). The positive effects of the revised milk and cheese allowances in the special supplemental nutrition program for women, infants, and children. J. Acad. Nutr. Diet..

[35] Andreyeva T., Luedicke J. (2013). Federal food package revisions: Effects on purchases of whole-grain products. Am. J. Prev. Med..

[36] Andreyeva T., Luedicke J., Tripp A.S., Henderson K.E. (2013). Effects of reduced juice allowances in food packages for the women, infants, and children program. Pediatrics.

[37] Weinfield N.S., Borger C., Au L.E., Whaley S.E., Berman D., Ritchie L.D. (2020). Longer Participation in WIC Is Associated with Better Diet Quality in 24-Month-Old Children. J. Acad. Nutr. Diet..

[38] Guthrie J.F., Catellier D.J., Jacquier E.F., Eldridge A.L., Johnson W.L., Lutes A.C., Anater A.S., Quann E.E. (2018). WIC and non-WIC Infants and Children Differ in Usage of Some WIC-Provided Foods. J. Nutr..

[39] United States Department of Agriculture (2016). WIC Food Packages-Maximum Monthly Allowances. https://www.fns.usda.gov/wic/wic-food-packages-maximum-monthly-allowances.

[40] National Academies of Sciences, Engineering and Medicine (2017). Review of WIC Food Packages: Improving Balance and Choice: Final Report.

[41] Keim S.A., Branum A.M. (2015). Dietary intake of polyunsaturated fatty acids and fish among US children 12–60 months of age. Matern. Child. Nutr..

[42] Fiocchi A., Assa’ad A., Bahna S. (2006). Food allergy and the introduction of solid foods to infants: A consensus document. Adverse Reactions to Foods Committee, American College of Allergy, Asthma and Immunology. Ann. Allergy Asthma Immunol..

[43] Hamner H.C., Perrine C.G., Gupta P.M., Herrick K.A., Cogswell M.E. (2017). Food Consumption Patterns among U.S. Children from Birth to 23 Months of Age, 2009–2014. Nutrients.

[44] Davis K.E., Li X., Adams-Huet B., Sandon L. (2018). Infant feeding practices and dietary consumption of US infants and toddlers: National Health and Nutrition Examination Survey (NHANES) 2003–2012. Public Health Nutr..

[45] Santiago-Torres M., Kratz M., Lampe J.W., Tapsoba J.D.D., Breymeyer K.L., Levy L., Villaseñor A., Wang C.-Y., Song X., Neuhouser M.L. (2016). Metabolic responses to a traditional Mexican diet compared with a commonly consumed US diet in women of Mexican descent: A randomized crossover feeding trial. Am. J. Clin. Nutr..

[46] Mattei J., Sotres-Alvarez D., Daviglus M.L., Gallo L.C., Gellman M., Hu F.B., Tucker K.L., Willett W.C., Siega-Riz A.M., van Horn L. (2016). Diet Quality and Its Association with Cardiometabolic Risk Factors Vary by Hispanic and Latino Ethnic Background in the Hispanic Community Health Study/Study of Latinos. J. Nutr..

